# Glucose Interference in Urine Biomarkers and Implications for Sodium-Glucose Cotransporter 2 Inhibition

**DOI:** 10.1016/j.ekir.2025.09.009

**Published:** 2025-09-09

**Authors:** Greco B. Malijan, Daniel Chapman, Stewart Moffat, Rebecca J. Sardell, Natalie Staplin, Martin J. Landray, Colin Baigent, Michael G. Shlipak, Richard Haynes, Joachim H. Ix, Michael Hill, William G. Herrington, Parminder K. Judge

**Affiliations:** 1 Renal Studies Group, Clinical Trial Service Unit and Epidemiological Studies Unit, Nuffield Department of Population Health, University of Oxford, Oxford, UK; 2 Wolfson Laboratory, Clinical Trial Service Unit and Epidemiological Studies Unit, Nuffield Department of Population Health, University of Oxford, Oxford, UK; 3 Kidney Health Research Collaborative, San Francisco Veterans Affairs Medical Center and University of California San Francisco, San Francisco, California, USA; 4 Oxford Kidney Unit, Oxford University Hospitals NHS Foundation Trust, Oxford, UK; 5 Division of Nephrology and Hypertension, Department of Medicine, University of California San Diego, San Diego, California, USA

**Keywords:** glycosuria, human cartilage glycoprotein-40, interference, interleukin-18, sodium-glucose cotransporter-2 inhibitor

## Introduction

Several urine biomarkers reflecting kidney tubular health and disease have been developed and may provide additional prognostic information alongside estimated glomerular filtration rate and urine albumin-to-creatinine ratio.^1^^,^^2^ Urine tubular biomarkers such as dickkopf-3, interleukin-18 (IL-18), kidney injury molecule-1, monocyte chemoattractant protein-1, neutrophil gelatinase-associated lipocalin, and human cartilage glycoprotein-40 (YKL-40) have been investigated in the context of acute kidney injury, kidney function decline, kidney drug monitoring, and tubulointerstitial fibrosis. Other biomarkers such as alpha-1-microglobulin, epidermal growth factor, and uromodulin have been studied for their proposed roles reflecting tubular reserve and function.

Sodium-glucose cotransporter 2 (SGLT2) inhibitors have been shown to reduce risk of kidney disease progression, risk of heart failure, and risk of adverse events of acute kidney injury.^3^ They are recommended for use in adults with chronic kidney disease (CKD)^4^ and widely prescribed. SGLT2 inhibition markedly increases urine glucose excretion, and although glycosuria is attenuated when estimated glomerular filtration rate is decreased, substantial glycosuria remains evident even in moderate-to-severe CKD.^5^ This is important to consider because there is experimental and clinical interest in using urine kidney tubule biomarkers; however, such glycosuria can interfere with laboratory assays. Previous work has illustrated how glycosuria importantly interferes with the Jaffe reaction used to measure creatinine and account for urine tonicity in spot urine samples.^6^ Glucose interference has also been identified in other urine sample assays (e.g., uric acid, urea, and total protein).^7^

We were unable to identify published information on any glucose interference on urine biomarker assays of interest for the EMPA-KIDNEY trial,^8^ so we aimed to assess whether alpha-1-microglobulin, measured using nephelometry; and dickkopf-3, epidermal growth factor, IL-18, kidney injury molecule-1, monocyte chemoattractant protein-1, neutrophil gelatinase-associated lipocalin, uromodulin, and YKL-40, measured using multiplex electrochemiluminescence immunoassays, are affected by glycosuria. We used urine samples from a cohort of adults with CKD with appropriate ethics approvals. Each urine sample was divided into 3 aliquots, with 1 serving as control and the other 2 being spiked with glucose to reach effective concentrations of 28 mmol/l and 111 mmol/l, which approximately correspond to the interquartile range of urine glucose concentrations among patients treated with SLGT2 inhibitors. Detailed laboratory and statistical methods are provided in the Supplementary Methods, including assay characteristics summarized in Supplementary Table S1.

## Results

Urine samples from 139 participants with CKD were analyzed. At baseline, the mean ± SD age of participants was 64 ± 13 years; 37 were female (27%), and 36 had diabetes (26%) (Supplementary Table S2). The mean ± SD estimated glomerular filtration rate was 36.5 ± 11.2 ml/min per 1.73 m^2^. The median (Q1–Q3) urine albumin-to-creatinine ratio was 52 (16–131) mg/mmol, with 15 (11%), 30 (22%), and 94 (68%) having urine albumin-to-creatinine ratio levels at < 3, ≥ 3 to < 30, and ≥ 30 mg/mmol, respectively. No participants were taking an SGLT2 inhibitor.

**Figure 1:**
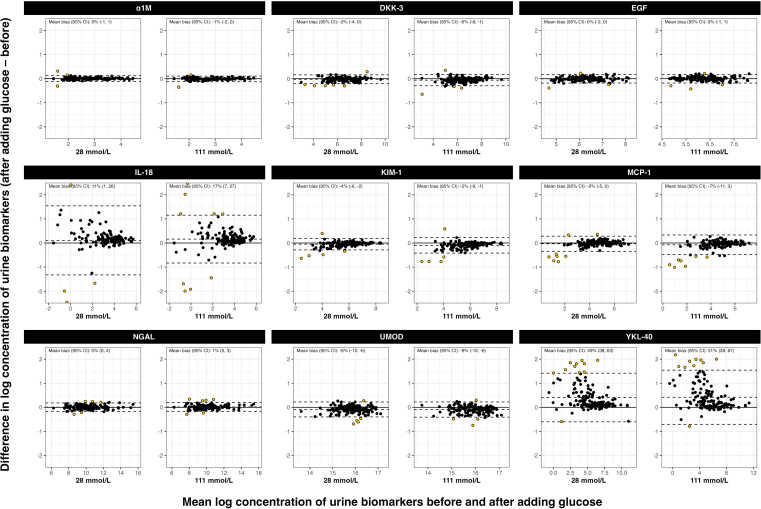
Bland-Altman plots for urine tubular biomarkers, by glucose concentration (28 mmol/l vs. control and 111 mmol/l vs. control). The x-axis indicates the mean log (base e) concentration of urine biomarkers before and after adding glucose (i.e., indicated glucose concentration vs control). The y-axis indicates the difference in log (base e) concentration of urine biomarkers after adding glucose minus before adding glucose. Mean bias and 95% CI are shown in text within each plot. Mean bias values have been back-transformed from natural log scale onto the original scale to give a percentage difference. The 95% limits of agreement are shown as dashed black lines. Points in yellow indicate measurement comparisons outside the limits of agreement. α1M, alpha-1 microglobulin; CI, confidence interval; DKK-3, dickkopf-3; EGF, epidermal growth factor; IL-18, interleukin-18; KIM-1, kidney injury molecule-1; MCP-1, monocyte chemoattractant protein-1; NGAL, neutrophil gelatinase-associated lipocalin; UMOD, uromodulin; YKL-40, human cartilage glycoprotein-40.

Bland-Altman plot analyses showed large positive mean bias at 28 mmol/l glucose versus control for 2 analytes. IL-18 had a mean bias of 11% (95% confidence interval: 1%–26%) and YKL-40 a 49% mean bias (38%–63%). Results were comparable at glucose concentration 111 mmol/l (Figure 1). There was a modest negative observed mean bias at 28 mmol/l glucose concentration versus control for dickkopf-3 (−2%, −4% to 0%), kidney injury molecule-1 (−4%, −6% to −2%), and uromodulin (−8%, −12% to −6%). The estimated mean bias values were similar at 111 mmol/l glucose concentration for these biomarkers. There was no evidence of any bias in measurements of alpha-1-microglobulin, epidermal growth factor, monocyte chemoattractant protein-1, and neutrophil gelatinase-associated lipocalin.

## Discussion

In this study evaluating the impact of glycosuria on urine tubular biomarker measurements, we found that IL-18 and YKL-40 measurements have a substantial positive bias in the presence of the amount of urinary glucose expected during use of SGLT2 inhibitors. There were approximately 11% and 49% overestimations of IL-18 and YKL-40, respectively. There were small negative biases for dickkopf-3, kidney injury molecule-1, and uromodulin; and no significant effects on epidermal growth factor, monocyte chemoattractant protein-1, and neutrophil gelatinase-associated lipocalin measured using electrochemiluminescence immunoassays. There was no meaningful interference for alpha-1-microglobulin.

These findings have important implications given the expanding use of SGLT2 inhibitors in CKD management and the growing interest in urine tubular biomarkers for risk stratification and monitoring kidney health. Glucose interference likely occurs through sample matrix changes that disrupt antigen-antibody interactions and protein glycation at antigen recognition sites, which may be present in IL-18 and YKL-40. However, predicting which analytes are most susceptible to glycation requires detailed structural knowledge of epitopes that is not routinely available.^9^

Key strengths of the study are the use of glucose concentrations reflecting the physiologic range observed with SGLT2 inhibitor therapy and evaluation of multiple biomarkers using validated assay platforms. Limitations of the study include the *in vitro* nature of the experiments, which may not fully capture *in vivo* conditions. For example, we did not evaluate potential further interference from other analytes that may be altered with SGLT2 inhibitor use such as ketone bodies or pH, for which reported changes are generally modest (Supplementary References S1-S5).

In summary, glycosuria substantially interferes with electrochemiluminescence immunoassays for IL-18 and YKL-40. There is a need for increased awareness of the potential for glycosuria to affect urine assays, and glucose interference experiments should be standard practice when developing any urine biomarker assays in the era of SGLT2 inhibitors.

## Disclosure

GBM, DC, SM, RS, NS, MJL, CB, RH, MH, WGH, and PKJ report institutional grant funding from Boehringer Ingelheim and Eli Lilly for the EMPA-KIDNEY trial. NS reports institutional grant funding from Novo Nordisk. RH reports institutional grant funding from Novartis and trial drug supply from Roche and Regeneron. CB reports grant funding from the Medical Research Council, National Institute for Health and Care Research (NIHR) Health Technology Assessment (HTA) (17/140/02) and Health Data Research UK; and advisory roles for Merck, NIHR HTA, the British Heart Foundation, and the European Society of Cardiology. WGH reports advisory roles for the UK Kidney Association, European Renal Association, European Society of Cardiology, and KDIGO. MGS reports institutional grant funding from Bayer and receipt of honoraria from AstraZeneca, Bayer, and Boehringer Ingelheim. JHI reports investigator-initiated research grant support from Breakthrough T1D and receipt of honoraria from AstraZeneca, Alpha Young, and Bayer.

## Supplementary Material

Supplementary File (PDF) **Supplementary Methods. Supplementary References. Table S1.** Characteristics of assays used in the experiment. **Table S2.** Baseline characteristics of participants with urine samples included in the laboratory interference experiment.

## References

[1] Ix J.H., Shlipak M.G. (2021). The promise of tubule biomarkers in kidney disease: a review. Am J Kidney Dis.

[2] Schrauben S.J., Zhang X., Xie D. (2025). Urine biomarkers for diabetic kidney disease progression in participants of the chronic renal insufficiency cohort study. Clin J Am Soc Nephrol.

[3] Nuffield Department of Population Health Renal Studies Group (2022). SGLT2 inhibitor Meta-Analysis Cardio-Renal Trialists’ Consortium. Impact of diabetes on the effects of sodium glucose co-transporter-2 inhibitors on kidney outcomes: collaborative meta-analysis of large placebo-controlled trials. Lancet.

[4] Kidney Disease: Improving Global Outcomes (KDIGO) CKD Work Group (2024). KDIGO 2024 clinical practice guideline for the evaluation and management of chronic kidney disease. Kidney Int.

[5] Hu S., Lin C., Cai X. (2022). The urinary glucose excretion by sodium–glucose cotransporter 2 inhibitor in patients with different levels of renal function: a systematic review and meta-analysis. Front Endocrinol.

[6] Chapman D., Judge P.K., Sardell R.J. (2023). Interference of urinary albumin-to-creatinine ratio measurement by glycosuria: clinical implications when using SGLT-2 inhibitors. Kidney Int.

[7] Mašković S., Nikolac Gabaj N. (2025). Ascorbic acid and glucose can cause significant interference on quantitative measurement of biochemistry analytes in urine. Lab Med.

[8] EMPA-KIDNEY Collaborative Group, Herrington W.G., Staplin N. (2023). Empagliflozin in patients with chronic kidney disease. N Engl J Med.

[9] Mo J., Jin R., Yan Q., Sokolowska I., Lewis M.J., Hu P. (2018). Quantitative analysis of glycation and its impact on antigen binding. mAbs.

