# Supplementary material for: Glucose Interference in Urine Biomarkers and Implications for Sodium-Glucose Cotransporter 2 Inhibition
Source: Kidney Int Rep. 2025 Sep 9;10(11):4090–3. doi: 10.1016/j.ekir.2025.09.009 (PMC12640033; doi:10.1016/j.ekir.2025.09.009)
Supplement: Supplementary File (PDF) — Supplementary Methods. Supplementary References. Table S1. Characteristics of assays used in the experiment. Table S2. Baseline characteristics of participants with urine samples included in the laboratory interference experiment. [file mmc1.pdf]

Glucose interference in urine biomarkers and implications for SGLT2 inhibition

Supplementary Material

Contents

Supplementary Methods **2**
Laboratory Methods 2
Statistical Analyses 2
Supplementary Tables **3**
Supplementary Table S1. Characteristics of assays used in the experiments 4
Supplementary Table S2. Baseline characteristics of participants with urine samples included in the laboratory interference experiment 5
Supplementary References **6**

Supplementary Methods

Early morning urine samples collected and stored as part of a CKD trial were used for laboratory interference experiments. Urine samples were stored long-term at -80°C until analysis. 139 participants' samples with a urine volume of at least 1ml were used for this study.

Laboratory Methods

Each urine sample was thawed, mixed by inversion, and separated into three 245µl aliquots. One aliquot had 70µl of deionized water added (control sample), and the remaining aliquots were spiked with 70µl of either 22.5 or 90 g/l of glucose. The resulting spiked glucose concentrations were 28 mmol/l and 111 mmol/l, respectively. These concentrations correspond to the approximate first and third quartiles of the urine glucose levels in the empagliflozin group at 18 months in EMPA-KIDNEY, a large randomized trial of empagliflozin versus placebo among adults with CKD.[1] Each aliquot was incubated at 37°C for two hours to simulate physiologic conditions prior to analysis. Urine alpha-1 microglobulin concentrations were determined as single measurements using the Atellica Nephelometer 630 analyzer (Siemens Healthineers, Erlangen, Germany). Immunoassays for urine dickkopf-3, epidermal growth factor, interleukin-18, kidney injury molecule-1, monocyte chemoattractant protein-1, neutrophil gelatinase-associated lipocalin, uromodulin, and human cartilage glycoprotein-40 were developed in-house in multiplex panels using reagents from MesoScale Discovery on the MESO QuickPlex SQ 120 platform (MesoScale Diagnostics, Rockville, MA, USA). Sample reformatting, dilution, and electrochemiluminescence analyses were carried out using the Biomek i7-A015 Automated Liquid Handler system (Beckman Coulter, Brea, CA, USA). Multiplex panel measurements were conducted in duplicates to minimize measurement errors. All sample storage, pre-analytical preparations, assay validation, optimization, and measurements were conducted in the Wolfson Laboratory located at the University of Oxford (following the Clinical & Laboratory Standards Institute guidelines).

Statistical Analyses

Biomarker values were log-transformed (i.e., natural log) to address skewness of biomarker concentration. Bland-Altman plots comparing the mean and difference of paired measurements of urine specimens spiked with glucose versus control were constructed using log-transformed biomarker values. Mean bias with 95% confidence interval (CI) and limits of agreement (LOA) were presented. Because biomarker values were log-transformed, mean bias values have been back-transformed onto the original scale to give a percentage difference. All statistical programming were performed using R version 4.4.2 (R Foundation for Statistical Computing, Vienna, Austria) and SAS version 9.4 (SAS Institute, Cary, NC, USA).

[1] EMPA-KIDNEY Collaborative Group. Empagliflozin in Patients with Chronic Kidney Disease. *New England Journal of Medicine*. Published online November 4, 2022:1-11. doi:10.1056/NEJMoa2204233

Supplementary Tables

Supplementary Table S1. Characteristics of assays used in the experiments

Urine Assay	Dilution Factor	Concentration*	Between-plate Coefficient of Variation (%)	Limits of Detection*
**Nephelometry**				
Alpha-1 microglobulin	1	28.4	1.3	5.34-171
**Electrochemiluminescence Assays (Multiplex Panels)**				
Dickkopf-3	1	6683	8.6	10.7-44000
Epidermal growth factor	49	11.7	5.7	0.2-49000
Interleukin-18	1	512	4.8	30-12700
Kidney injury molecule-1	1	2231	2.6	19.5-20000
Monocyte chemoattractant protein-1	1	624	4.3	35-6400
Neutrophil gelatinase-associated lipocalin	49	19300	14.1	8.4-1681680
Uromodulin	49	49637	7.1	1465-294000000
Human cartilage glycoprotein-40	1	1418	7.8	37-18160

*Concentration and limits of detection reported with mg/l for alpha-1 microglobulin and pg/ml for the biomarkers measured using multiplex panels; concentration refers to the biomarker levels at which the coefficients of variation were determined

Supplementary Table S2. Baseline characteristics of participants with urine samples included in the laboratory interference experiment

Characteristic	Included (N=139)
**DEMOGRAPHICS**	
Age at randomization, years	64±13
Female sex, no. (%)	37 (26.6)
Race, no. (%)†	
Black	3 (2.2)
South Asian	1 (0.7)
Other	5 (3.6)
White	130 (94.5)
History of diabetes, no. (%)‡	36 (25.9)
Systolic	146±16
Diastolic BP	82±11
Body mass index, kg/m^2	30±5
**LABORATORY MEASUREMENTS**	
Estimated glomerular filtration rate, ml/min/1.73 m^2	
Mean ± SD	36.5±11.2
Distribution, no. (%)	
<30	47 (33.8)
≥30 to <45	59 (42.5)
≥45	32 (23.1)
Urine albumin-to-creatinine ratio, mg/mmol	
Median [Q1, Q3]	52 [16, 131]
Distribution, no. (%)	
<3	15 (10.8)
3 to 30	30 (21.6)
>30	94 (67.6)
RAS inhibitor use at baseline, no. (%)	116 (83.5)

* Plus-minus values are means ± SD. 2 participants had missing body mass index measurements, and 1 participant had missing estimated glomerular filtration rate at baseline.

† Race was reported by the patients. The “other” category indicates that the race was not specified or the patient preferred not to answer.

‡ History of diabetes was defined as patient-reported history of diabetes of any type, use of glucose-lowering medication, or a glycated haemoglobin level of at least 48 mmol/mol at the randomization visit.

Supplementary References

S1. Ferrannini E, Baldi S, Frascerra S, et al. Renal Handling of Ketones in Response to Sodium–Glucose Cotransporter 2 Inhibition in Patients With Type 2 Diabetes. *Diabetes Care*. 2017;40(6):771-776. doi:10.2337/dc16-2724

S2. Scarr D, Lovblom E, Ye H, et al. Ketone production and excretion even during mild hyperglycemia and the impact of sodium-glucose co-transporter inhibition in type 1 diabetes. *Diabetes Res Clin Pract*. 2024;207:111031. doi:10.1016/j.diabres.2023.111031

S3. Harmacek D, Pruijm M, Burnier M, et al. Empagliflozin Changes Urine Supersaturation by Decreasing pH and Increasing Citrate. *J Am Soc Nephrol JASN*. 2022;33(6):1073-1075. doi:10.1681/ASN.2021111515

S4. Biancalana E, Rossi C, Raggi F, et al. Empagliflozin and Renal Sodium-Hydrogen Exchange in Healthy Subjects. *J Clin Endocrinol Metab*. 2023;108(8):e567-e573. doi:10.1210/clinem/dgad088

S5. Sen T, Heerspink HJL. A kidney perspective on the mechanism of action of sodium glucose co-transporter 2 inhibitors. *Cell Metab*. 2021;33(4):732-739. doi:10.1016/j.cmet.2021.02.016
